# Association between rheumatoid arthritis and thyroid dysfunction: A meta-analysis and systematic review

**DOI:** 10.3389/fendo.2022.1015516

**Published:** 2022-10-13

**Authors:** Yi-jing Liu, Hai-bing Miao, Shu Lin, Zhen Chen

**Affiliations:** ^1^ Department of Rheumatology and Immunology, The Second Affiliated Hospital of Fujian Medical University, Quanzhou, China; ^2^ Centre of Neurological and Metabolic Research, The Second Affiliated Hospital of Fujian Medical University, Quanzhou, China; ^3^ Group of Neuroendocrinology, Garvan Institute of Medical Research, Sydney, NSW, Australia

**Keywords:** rheumatoid arthritis, subclinical hyperthyroidism, subclinical hypothyroidism, thyroid dysfunction, immune system

## Abstract

**Objective:**

Rheumatoid arthritis (RA) is an autoimmune disorder. Multiple studies have investigated the risk of thyroid dysfunction in patients with RA but have reached conflicting conclusions. This systematic review aimed to determine whether patients with RA are at higher risk of thyroid dysfunction.

**Methods:**

We comprehensively reviewed online literature databases, including PubMed, Scopus, Embase, and the Cochrane Library, from their respective inception dates to March 25, 2022. Studies that provided data on at least one case of thyroid dysfunction in RA patients and their controls were included. Based on these data, we calculated pooled odds ratios (ORs) and their corresponding 95% confidence intervals (CIs) for thyroid dysfunction in RA and non-RA patients.

**Results:**

Twenty-nine studies met the inclusion criteria, involving a total of 35,708 patients with RA. The meta-analysis showed that, compared with non-RA patients, RA patients had an increased risk of developing thyroid dysfunction, particularly hypothyroidism (OR 2.25, 95% CI 1.78–2.84). Subgroup analysis suggested that study type and sample source of control group were the source of heterogeneity.

**Conclusions:**

Patients with RA are at increased risk of developing thyroid dysfunction, especially hypothyroidism. Routine biochemical examination of thyroid function in RA patients should be strengthened. Larger prospective studies are needed to explore the causal relationship between RA and thyroid dysfunction, and to investigate the impact of thyroid dysfunction on RA disease activity, drug efficacy, and medication safety.

**Systematic review registration:**

https://www.crd.york.ac.uk/prospero/, identifier CRD42022331142.

## Introduction

Rheumatoid arthritis (RA) is an autoimmune disease characterized by systemic inflammation, persistent synovitis, and autoantibodies (particularly against citrullinate peptides and rheumatoid factors) (1). With repeated episodes of joint inflammation, the normal structure of the joint is destroyed, ultimately resulting in reduced mobility and increased disability (2). In addition, many RA patients also present with extra-articular clinical symptoms (3). Recently, a meta-analysis (4) determined that the global prevalence of RA was approximately 0.46% over the past 40 years. Moreover, RA has been found to be potentially associated with a variety of autoimmune diseases, including thyroid dysfunction (5). Thyroid dysfunction mainly includes hyperthyroidism, hypothyroidism, subclinical hyperthyroidism and subclinical hypothyroidism. Autoimmune thyroid disease (AITD), which mainly includes Hashimoto’s thyroiditis and Graves’ disease, is the most common cause of various thyroid dysfunction and is manifested by the production of antibodies against thyroid peroxidase, thyroglobulin or thyrotropin receptor autoantigens (6, 7). In addition to clinical manifestations, laboratory tests are more important in the definition of several thyroid diseases. Common test indicators include thyroid-stimulating hormone (TSH), triiodothyronine (T3), thyroxine (T4), free triiodothyronine (fT3), free thyroxine (fT4) (8). The range of reference values given by different experimental equipment and detection kits varies to a certain extent, but the trend of indicator change has been widely recognized (9, 10). Hypothyroidism and hyperthyroidism can be divided into overt and subclinical stages. Based on hormone levels, hyperthyroidism and hypothyroidism are defined as excessive and insufficient thyroid hormones, respectively (11). Most thyroid diseases and rheumatoid arthritis are chronic diseases, and unfortunately, early signs of thyroid dysfunction are not specific and are often overshadowed by clinical manifestations of other diseases in the absence of biochemical tests of thyroid function (12). Thyroid dysfunction can be detrimental to health and, if left undiagnosed or treated, can have serious adverse consequences and, in exceptional cases, can be fatal (12).

Findings from studies on the risk of thyroid dysfunction in RA patients are conflicting (13). For example, a study by McCoy et al. (14) concluded that RA patients did not have significantly increased risk of hypothyroidism as compared with non-RA patients (14). Graves’ disease is the most common cause of hyperthyroidism and has a high comorbidity rate with RA (15). Considering the accumulation of evidence, we conducted a systematic review and meta-analysis to assess whether patients with RA are at a higher risk of the four major thyroid dysfunctions. We also used subgroup analysis to investigate the influence of control group origin, study type, regions, and publication date on the results of the overall analysis, as well as to explore sources of heterogeneity.

## Methods

### Search strategy

A systematic review and a meta-analysis were conducted according to the Preferred Reporting Items for Systematic Reviews and Meta-analysis (PRISMA) guidelines (16). We searched PubMed, Scopus, Embase, and the Cochrane Library databases, for all eligible studies, regardless of language, from their respective inception dates to 25 March 2022. The search terms used were “Thyroid Dysfunction,” “Thyroid Diseases,” “Hypothyroidism,” “Hyperthyroidism,” and “Arthritis, Rheumatoid” (please see appendix **eTable 1** for the complete search strategy). This study is registered on PROSPERO, number CRD42022331142.

### Inclusion and exclusion criteria

A Population, Intervention, Comparator, Outcome and Study design (PICOS) scheme was used to clarify our research objectives. Our meta-analysis was designed to answer the question: does rheumatoid arthritis increase the risk of thyroid dysfunction? Representatives of the PICOS regimen as follows: patients with RA (P); comparison with non-RA, non-systemic lupus erythematosus (SLE), or healthy subjects (C); the diagnosis of thyroid dysfunction (O); observational studies (S). In our meta-analysis, patients with thyroid dysfunction mainly included hyperthyroidism, hypothyroidism, subclinical hypothyroidism, and subclinical hyperthyroidism.

Studies that up to the following standards were incorporated in this meta-analysis: Observational studies that included RA and non-RA controls provided the number of samples in both groups and provided the number or prevalence of thyroid dysfunction in each group. Patients who met the American College of Rheumatology diagnostic criteria for RA or the European League Against Rheumatism classification criteria, or who had at least one documented rheumatoid arthritis diagnosis in the medical records of hospitals and medical databases, were included in the study. In addition, to be included in this study, at least one case of hypothyroidism, subclinical hypothyroidism, hyperthyroidism, or subclinical hyperthyroidism had to be detected in RA patients and/or controls. To test whether the findings are common across countries, we did not restrict the language used to publish the articles.

We excluded reviews, editorials, case reports, comments, books, etc. Studies without available abstracts or complete manuscripts were also excluded. If there was duplication in the study population among studies, only the latest article was included. Given that a strong association between thyroid disease and systemic lupus erythematosus has been reported for more than 50 years (17), studies with SLE patients as controls were also excluded.

### Data extraction and quality assessment

Two reviewers (Y.L. and H.M.) independently fetched the data from eligible papers and then combined the data. Disagreements were settled through discussion to consensus. We extracted the following basic information and outcome-related data from studies that up to our inclusion criteria: year of publication, the name of the first author, nationality of the author, average duration of RA, study design, the number of cases and controls, the mean age and sex ratio among RA and non-RA patients, methodological quality of the study, and number of people with thyroid dysfunction. We focused on the statistical results of the four types of thyroid dysfunction in the RA group and the control groups. We preferentially extracted the raw data in the study report, and if the study did not provide the raw data, the prevalence value provided by the study was used for calculation. We used the Newcastle-Ottawa Scale (NOS) to assess the quality of the included literature (18). The overall score is 9, with 7-9 being high quality, 4-6 being medium quality, and 0-3 being low quality.

### Statistical analysis

The association strength between RA and four types of thyroid dysfunction was calculated, expressed by the odds ratio (OR), with the corresponding 95% confidence interval (CI), using the sample sizes of RA and control groups as well as the statistical results of four types of thyroid dysfunction. OR > 1 indicates that RA increases the risk of thyroid dysfunction, OR = 1 indicates that there is no correlation between RA and thyroid dysfunction, and OR < 1 indicates that RA patients have a reduced risk of thyroid dysfunction.

P < 0.05 was recognized statistically significant. We used the I^2^ statistic and χ2 test to assess the heterogeneity of the pooled ORs (P < 0.1 considered statistically significant). I^2^ ≤ 25% indicated that heterogeneity was insignificant, I^2^ < 50% indicated low heterogeneity, I^2^ between 50% and 75% indicated medium heterogeneity, and I^2^ > 75% indicated high heterogeneity (19).

When the pooled OR was calculated, if I^2^ > 50%, the random effect model was used for sensitivity analysis and subgroup analysis; if I^2^ < 50%, the fixed-effect model was adopted (20). Egger’s test, Funnel plot, and Begg’s test were used to evaluate whether publication bias existed. P < 0.05 indicated that the detected publication bias was statistically significant, and the impact of potential publication bias was further assessed using the Duval and Tweedie non-parametric modified trim-and-fill method (21). All statistical analyses were accomplished using Stata V.16.0 software.

## Results

### Literature search results and study characteristics

**Figure 1:**
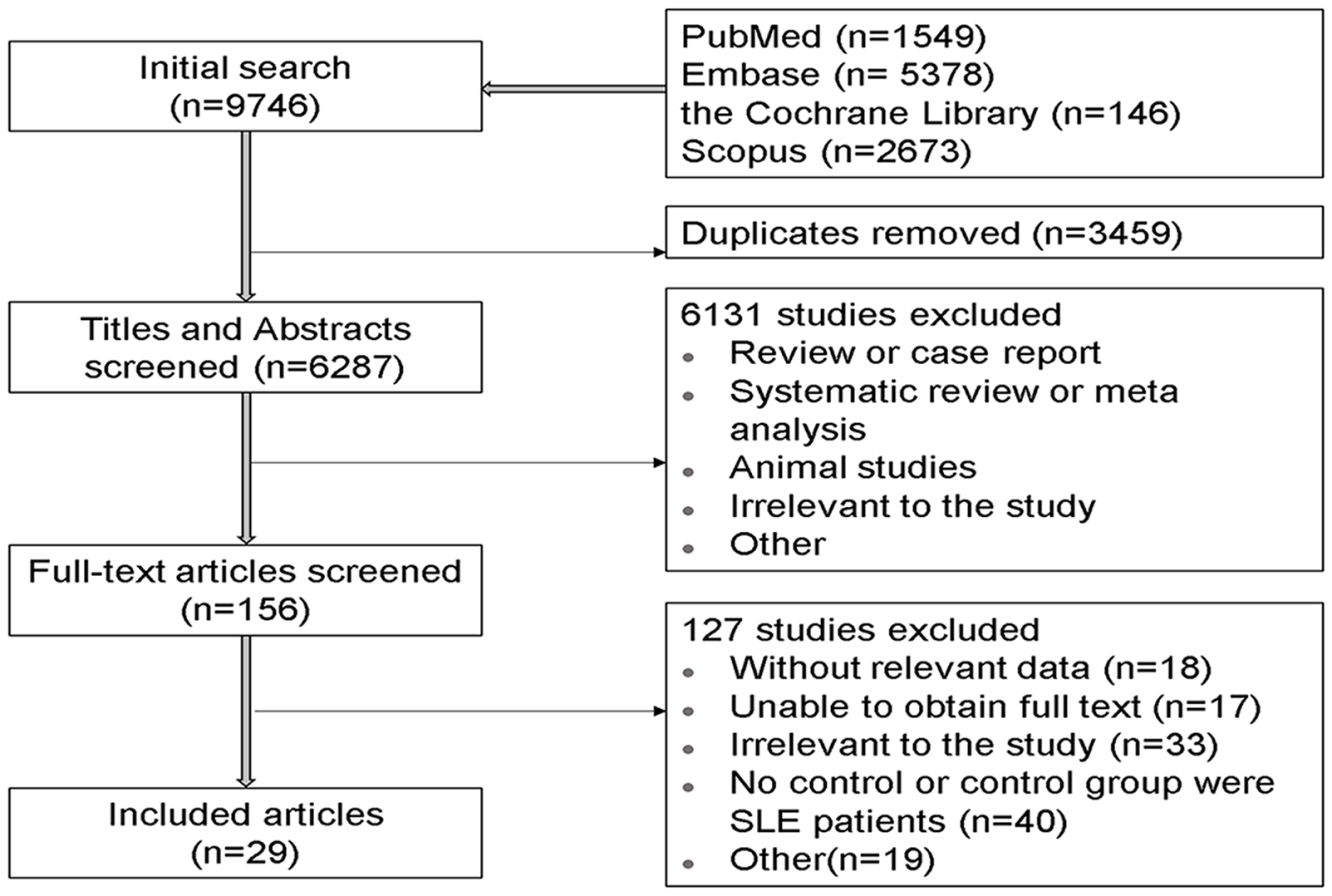
Flow chart of search results.

A preliminary search of four electronic databases, PubMed, Scopus, Embase, and the Cochrane Library, yielded 9,746 articles, including 1,549 PubMed articles, 5,378 Embase articles, 2,673 Scopus articles, and 146 Cochrane Library articles. After removing duplicates, 6,287 records remained. Two authors (Y.L. and H.M.) scanned the titles and abstracts of these articles as a preliminary screen and selected 156 articles that appeared eligible for inclusion. Further, the two authors then read the full text of these articles respectively. If there were disputes about inclusion of any of these papers, a third author (Z.C.) made the final decision. Finally, 29 studies (5, 11, 13, 14, 22–46) that met all inclusion criteria were absorbed in the meta-analysis. **Figure 1** shows the detailed screening steps.

**Table 1** describes the general characteristics of the literature included in our meta-analysis. Twenty-nine studies met the inclusion criteria, involving 35,708 RA patients and 149,421 non-RA controls. Among them, 15 studies (5, 11, 13, 22, 24, 25, 27, 31, 33, 34) provided (37–40, 45) prevalence values for hyperthyroidism, 12 provided (5, 11, 22, 24, 31, 33, 34, 36, 37, 42, 44, 45) prevalence values for subclinical hyperthyroidism, 26 provided (5, 11, 13, 14, 22–31, 33–41, 43, 45, 46) prevalence values for hypothyroidism, and 17 provided (5, 11, 14, 22, 24, 31–34, 36–39, 42–45) prevalence values for subclinical hypothyroidism. The NOS checklist was used to evaluate the quality of studies, and 7 studies (24.13%) assessed as high quality, and 18 studies (62.07%) identified as medium quality.

**Table 1:** Characteristic of basic information.

Author, year	Country	Total patients with RA	Age of RA(mean year ± SD)	Sex ratio of RA patients (M/F)	Duration of RA (mean year ± SD)	Source of control group	Number of control group	Study design	Quality score
E. Deseatnicova, 2022 (22)	Moldova	59	54.2 ± 9.6	NA	9.6 ± 5.6	Non-RA control	48	cross-section research	1
Nazary, K, 2021 (5)	Afghanistan	400	36 ± 08	159/241	0	Non-RA control	400	case-control study	7
Yadav, B., 2019 (23)	India	493	47 ± 12	68/425	3.97 ± 3.93	Osteoarthritis patients	165	cross-section research	5
Saqre, I. M., 2019 (24)	Egypt	60	41 ± 10.5	17/43	8.6 ± 4.1	Health control	60	case-control study	6
Li, Q., 2019 (11)	China	65	59.58 ± 11.64	13/52	7.12 ± 8.58	Health control	550	case-control study	8
Mahagna, H., 2018 (13)	Israel	11782	61.1 ± 17.0	2679/9103	NA	Non-RA control	57973	cross-section research	4
Huang, C. M., 2018 (25)	China	18267	53.6 ± 13.9	3945/14322	NA	Non-RA control	73068	case-control study	6
Guy, A., 2018 (26)	Israel	15	56.27 ± 13.35	1/14	NA	Health control	32	case-control study	3
Figueroa-Sánchez,2019 (27)	Mexico	78	42.75 ± 12.15	13/65	4.74 ± 6.04	Health control	81	cross-section research	6
El Achek, M. A., 2018 (28)	Tunisia	111	51.04[18-80]	17/94	6.54[0-29]	Ankylosing spondylitis patients	60	case-control study	2
Posselt, R. T., 2017 (29)	Brazil	210	53.7 ± 10.45	19/191	11.1 ± 8.9	Health control	141	cross-section research	6
Tascilar, K., 2016 (30)	Canada	1357	63.7 ± 9.4	539/818	3.26	Non-RA control	13570	case-control study	6
El-saadany, H., 2014 (31)	Egypt	40	29.1 ± 6.1	0/40	NA	Healthy female control	20	case-control study	5
Gomez, R., 2013 (32)	Colombia	86	56.4	9/77	NA	Arthropathy and fibromyalgia patients	86	case-control study	5
Acay, A., 2014 (33)	Turkey	80	48.09 ± 10.25	17/63	NA	Health control	122	cross-section research	4
McCoy, S. S., 2012 (14)	USA	650	55.8 ± 15.7	202/448	7.9 ± 5.2	Non-RA control	650	cohort study	7
Mousa, A. A., 2012 (34)	Egypt	217	36.3 ± 12.8	43/174	NA	Health control	120	case-control study	6
Fatima, F., 2010 (35)	India	800	46.15 ± 13.37	132/668	4.9 ± 5.50	Non-RA control	800	case-control study	5
Mobini, M., 2011 (36)	Iran	80	51.4 ± 12	4/76	NA	Osteoarthritis patients	80	case-control study	5
Przygodzka, M., 2009 (37)	Poland	100	54 ± 12.2	0/100	12.3	Non-RA control	55	case-control study	7
Haghighi, A., 2009 (38)	Iran	75	NA	5/70	NA	mechanical low back pain or osteoarthritis patients	66	case-control study	2
Al-Awadhi, A. M., 2008 (39)	Kuwait	177	38.3 ± 12.8	37/140	NA	Health control	577	cross-section research	5
Andonopoulos, A. P., 1996 (40)	Greece	101	57,9 ± 13,5	29/72	8.3 ± 7.7	Osteoarthritis patients	70	case-control study	7
Shiroky, J. B., 1993 (41)	Canada	119	NA	28/91	NA	Osteoarthritis or primary fibromyalgia patients	108	case-control study	7
Antonelli, A., 2006 (42)	Italy	91	59 ± 16	1/91	9 ± 7	Healthy female control	180	case-control study	6
Al-Awadhi, A. M., 1999 (43)	Kuwait	48	47 ± 11	5/43	NA	Non-inflammatory rheumatic diseases patients	90	case-control study	7
Innocencio, R. M., 2004 (44)	Brazil	25	49 ± 13	7/18	18 ± 11	Healthy blood donors	113	case-control study	4
Irena Kostic, 2006 (45)	Serbia	24	54 ± 9	3/21	8.54	Healthy blood donors	34	cross-section research	4
Maria José Santos, 2010 (46)	Portugal	98	49.2 ± 13.7	0/98	7.6	Healthy blood donors	102	case-control study	6

RA, rheumatoid arthritis; USA, The United States of America. NA, Not Available.

### Synthesis of results

#### RA and hyperthyroidism

Fifteen studies (5, 11, 13, 22, 24, 25, 27, 31) recorded the prevalence of hyperthyroidism in RA and control groups (33, 34, 37–40, 45). However, in 5 literatures (27, 31, 33, 34, 45), there is no information on hyperthyroidism in RA patients or controls.

**Figure 2:**
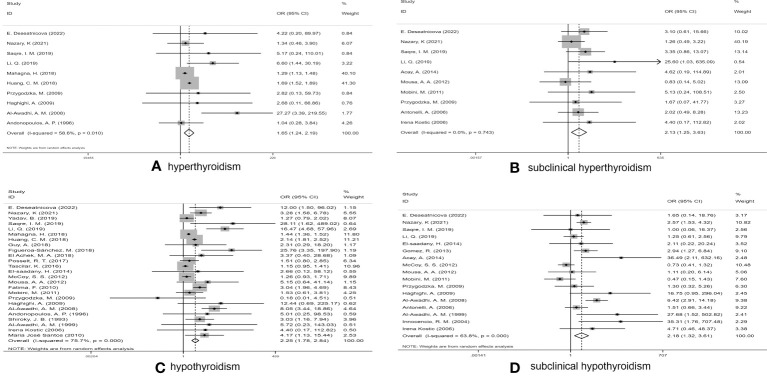
Forest plot for the risk of thyroid dysfunction among RA patients. **(A)** Forest plot for the risk of hyperthyroidism among RA patients, **(B)** Forest plot for the risk of subclinical hyperthyroidism among RA patients, **(C)** Forest plot for the risk of hypothyroidism among RA patients, **(D)** Forest plot for the risk of subclinical hypothyroidism among RA patients.

We executed a statistical analysis on the remaining 10 studies (**Figure 2A**). The risk of developing hyperthyroidism in RA patients was observably higher than in control groups (OR 1.65, 95% CI 1.24–2.19). Among the studies reporting statistically significant differences, some extremes occurred, such as in the study by AI-Awadhi et al. (39) suggested that RA patients had a higher risk of hyperthyroidism, while the study of Mahagna et al. (13) found that RA patients had a relatively low risk of hyperthyroidism.

#### RA and subclinical hyperthyroidism

After excluding two studies (31, 44), in which subclinical hyperthyroidism was not found in any subjects, the prevalence of subclinical hyperthyroidism in RA patients and controls was calculated in a total of 10 studies (5, 11, 22, 24, 33, 34, 36, 37, 42, 45) **(** **Figure 2B** **).** The pooled OR was 2.13 (95%CI 1.25–3.63) (P < 0.05), indicating that RA patients had more than twice the risk of subclinical hyperthyroidism as non-RA subjects, which was statistically significant. Due to low heterogeneity (I^2^ = 0.0%, P = 0.743), a fixed-effect model was used for statistical analysis.

#### RA and hypothyroidism

In the study conducted by Acay et al. (33), neither group included any cases of hypothyroidism. We thus conducted a combined analysis of the remaining 25 studies (5, 11, 13, 14, 22–28, 31, 34–41, 43, 45, 46) **(** **Figure 2C** **).** Due to the high I^2^ value (I^2^ = 75.7%), a random-effects model was applied. The pooled OR was 2.25 (95%CI 1.78–2.84) (P > 0.05). Our statistical results indicated that RA patients had a higher risk of hypothyroidism than of the other three types of thyroid dysfunction.

#### RA and subclinical hypothyroidism

A pooled analysis of 17 studies (5, 11, 14, 22, 24, 31–34, 36–39, 42–45) involving the association between RA and subclinical hypothyroidism was performed. The pooled OR was 2.18 (95% CI 1.32–3.61) (P < 0.05), indicating that the risk of subclinical hypothyroidism in RA patients was significantly higher than that in controls (**Figure 2D**), with an I2 value of 63.8% (P < 0.001); thus, we used a random effects model.

### Subgroup analyses

In addition to the pooled analysis of the risk of developing subclinical hyperthyroidism in patients with RA, there was significant heterogeneity in the pooled analysis of the other three types of thyroid dysfunctions. To further explore possible sources of heterogeneity and to determine whether specific factors influence the risk intensity for thyroid dysfunction among RA patients, we performed subgroup analyses by control group origin, study type, geographic region, and publication date. As shown in **Table 2**, the subgroup analysis showed that the origin of controls was the source of heterogeneity in the risk of hyperthyroidism, and the study type was the source of heterogeneity in the risk of subclinical hypothyroidism. Region and publication data did not significantly contribute to heterogeneity.

**Table 2:** Subgroup analysis of region, year of Publication, study design, and control group origin.

		Hyperthyroidism		Subclinical hyperthyroidism		Hypothyroidism		Subclinical hypothyroidism	
		OR, 95%CI	I^2^%, P	OR, 95%CI	I^2^%, P	OR, 95%CI	I^2^%, P	OR, 95%CI	I^2^%, P
Region	Europe	1.43(0.47,4.38)	0.00%, P=0.634	2.53(0.97,6.58)	0.00%, P=0.954	3.92(1.33,11.57)	14.2%, P=0.324	1.61(0.84,3.10)	0.00%, P=0.818
	Asia	1.68(1.22,2.30)	75.3%, P=0.001	1.91(0.85,4.27)	24.3%, P=0.265	2.54(1.82,3.54)	84.8%, P=0.000	3.17(1.31,7.70)	76.6%, P=0.000
	Africa	5.17(0.24,110.01)	–	2.09(0.72,6.05)	32.4%, P=0.224	5.51(1.64,18.58)	0.00%, P=0.620	1.31(0.39,4.48)	0.00%, P=0.885
	Americas	–	–	–	–	1.56(1.05,2.34)	69.0%, P=0.012	2.41(0.54,10.80)	83.3%, P=0.003
Year of Publication	2011–2022	1.56(1.21,2.02)	63.8%, P=0.017	2.11(1.17,3.83)	0.00%, P=0.453	1.84(1.45,2.33)	76.5%, P=0.000	1.49(0.86,2.60)	59.2%, P=0.009
	1991–2010	3.63(0.65,20.32)	57.0%, P=0.073	2.22(0.67,7.33)	0.00%, P=0.897	3.72(2.56,5.40)	4.40%, P=0.398	4.43(1.78,11.03)	56.5%, P=0.032
Study design	Case-control study	1.70(1.52,1.89)	0.00%, P=0.610	1.91(1.06,3.43)	0.00%, P=0.522	2.75(1.89,4.00)	71.3%, P=0.000	1.88(1.15,3.08)	45.7%, P=0.042
	Cross-section research	4.46(0.53,37.74)	77.2%, P=0.012	3.50(0.93,3.15)	0.00%, P=0.966	2.62(1.48,4.66)	78.6%, P=0.000	6.20(3.09,12.42)	0.00%, P=0.420
Control group origin	Non-RA control	1.48(1.23,1.79)	41.2%, P=0.116	1.78(0.85,3.75)	0.00%, P=0.696	1.76(1.42,2.19)	74.0%, P=0.000	1.76(0.86,3.61)	69.8%, P=0.002
	Health control	9.75(3.12,30.48)	0.00%, P=0.488	2.58(1.20,5.54)	0.00%, P=0.552	5.68(2.65,12.19)	59.0%, P=0.009	2.82(1.33,5.96)	57.2%, P=0.017

I^2^ indicates the level of heterogeneity. RA, rheumatoid arthritis.

### Sensitivity analysis

Sensitivity analysis was carried out by removing each of the studies consecutively to verify the reliability and stability of the pooled results, observe the effect of individual results on the overall analysis, and look for possible sources of heterogeneity.

**Figure 3:**
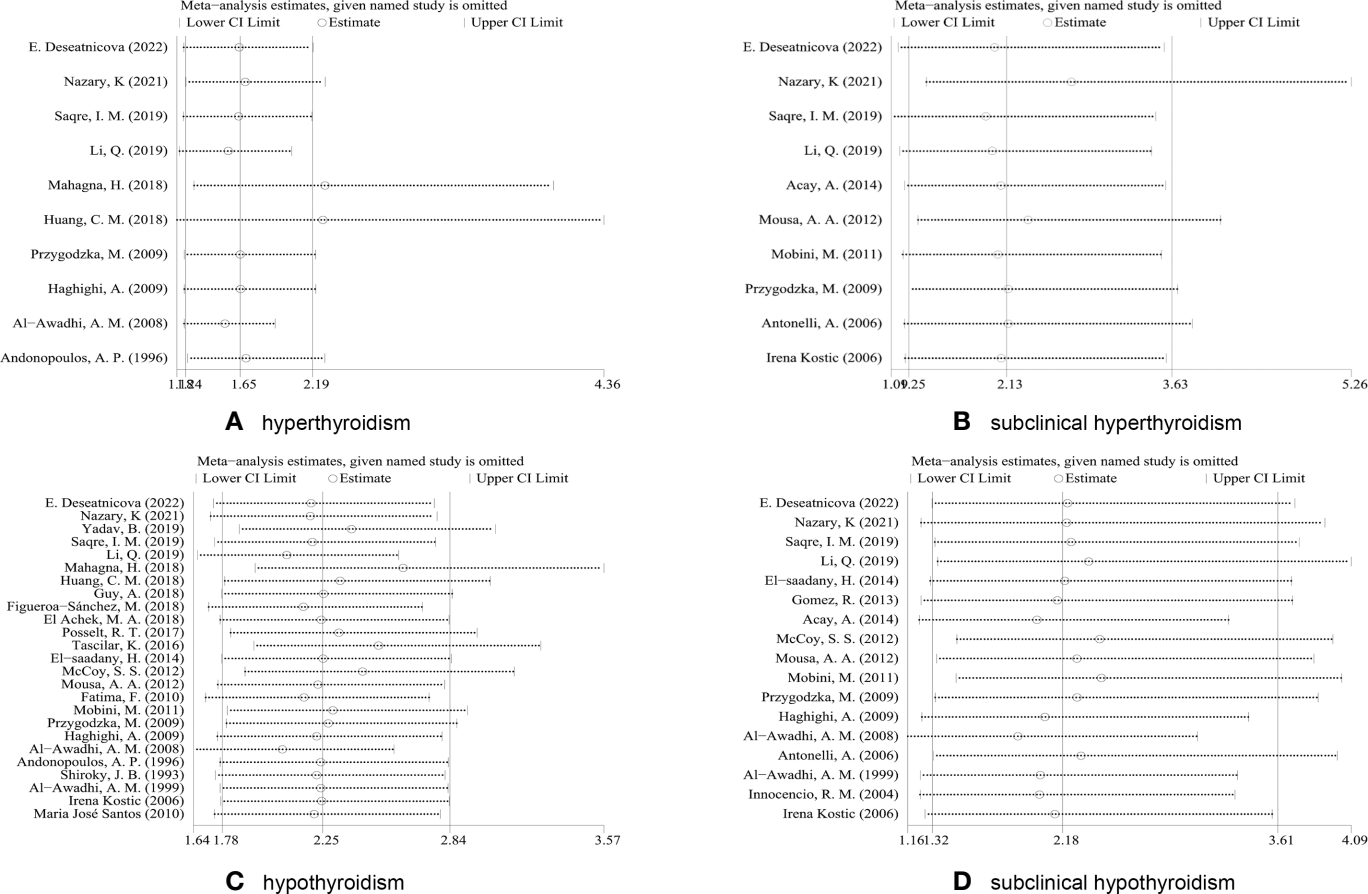
Sensitivity analysis based on excluding each study in turn. **(A)** Sensitivity analyses for the association of RA and hyperthyroidism. **(B)** Sensitivity analyses for the association of RA and subclinical hyperthyroidism. **(C)** Sensitivity analyses for the association of RA and hypothyroidism. **(D)** Sensitivity analyses for the association of RA and subclinical hypothyroidism.

Sensitivity analyses were performed for association of RA and four thyroid dysfunctions. If the pooled results suggested an increased risk of thyroid dysfunction even after exclusion of any study, the pooled analysis was considered robust. As shown in **Figure 3**, when sensitivity analysis was performed on the pooled results of studies related to subclinical hyperthyroidism, hypothyroidism, and subclinical hypothyroidism, the combined OR remained within the overall 95% CI range after the exclusion of any study, indicating that the statistical data were robust and reliable. However, in the sensitivity analysis of hyperthyroidism-related studies, the combined OR did not remain within the 95% CI range of the overall OR after excluding the studies of Mahagna et al. (13) or Huang et al. (25), and these two studies were considered to have an impact on the overall efficacy. Heterogeneity was significantly reduced after we excluded these two articles (P = 0.186, I^2^ = 30.3%). Therefore, the two studies were considered to represent a source of heterogeneity. Without the two studies, the combined OR of the remaining studies was 3.03 (95%CI 1.33–6.91, P = 0.008).

### Publication bias

**Figure 4:**
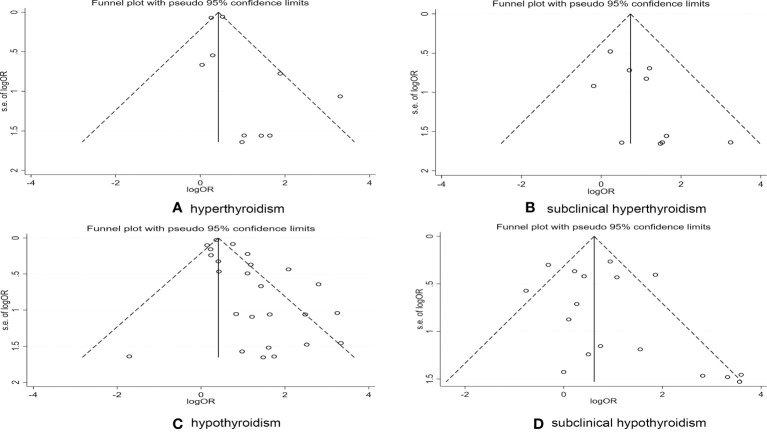
Publications bias detected by funnel plot on the risk of thyroid dysfunction in RA patients. **(A)** Publications bias detected by funnel plot on the risk of hyperthyroidism in RA patients. **(B)** Publications bias detected by funnel plot on the risk of subclinical hyperthyroidism in RA patients. **(C)** Publications bias detected by funnel plot on the risk of hypothyroidism in RA patients. **(D)** Publications bias detected by funnel plot on the risk of subclinical hypothyroidism in RA patients.

To detect whether publication bias existed, we created funnel plots, and conducted Begg’s and Egger’s tests. Typically, precise studies or studies with large numbers of subjects appear at the top of a funnel plot, while less precise studies or studies with fewer subjects appear at the bottom (21). As shown in **Figure 4**, these plots showed that there was no significant bias in these studies except for the abnormal funnel plot associated with RA and hypothyroidism **(** **Figure 4C** **).** The detection of publication bias was also confirmed in Egger’s test (**eTable 2**).

We attempted to evaluate the impact of publication bias on the aggregate results by making use of the trim-and-fill method (21). After four iterations, nine potential studies were added, and the results showed that publication bias had little impact on the stability of the results (**eFigure 1**).

## Discussion

This meta-analysis involved 35,708 RA patients and 149,421 control subjects. Our meta-analysis of 29 selected papers on the association between RA and thyroid dysfunction revealed a strong correlation between RA and thyroid dysfunction, particularly hypothyroidism. This yielded a pooled OR of 2.25 (95%CI 1.78–2.84) (**Figure 2**). In addition, RA was closely associated with subclinical hypothyroidism as well as subclinical hyperthyroidism, with ORs of 2.18 (95%CI 1.32–3.61) and 2.13 (95%CI 1.25–3.63), respectively, suggesting an increased risk of both thyroid dysfunctions more than doubled. The OR for hyperthyroidism among RA patients was 1.65 (95%CI, 1.24–2.19).

RA is an autoimmune inflammatory disease affecting symmetrical joints, usually characterized by persistent joint pain, tenderness, and joint destruction (47), while thyroid dysfunction is one of the most common chronic endocrine diseases (48, 49). Thyroid dysfunction mainly discussed in this meta-analysis included hyperthyroidism (low thyroid stimulating hormone [TSH], high free thyroxine [FT4]), hypothyroidism (high TSH, low FT4), subclinical hyperthyroidism (low TSH, normal FT4), subclinical hypothyroidism (high TSH, normal FT4) (49, 50). Because the clinical manifestations of thyroid disease are highly variable and usually nonspecific, the diagnosis of thyroid dysfunction relies primarily on laboratory biochemical tests (49). Although many studies have assessed the risk of thyroid dysfunction in patients with RA, their conclusions have been inconsistent, perhaps due to differences in the populations and geographical regions across studies (51). For example, Nazary et al. compared thyroid function in 400 newly diagnosed RA patients with matched controls and found a higher prevalence of thyroid dysfunction in RA patients (25.25% vs. 11.5%; P value = 0.00001). However, McCoy et al. (14) found that the incidence of hypothyroidism in RA patients was not significantly increased compared with non-RA patients (14). Unfortunately, many studies have not investigated these four types of thyroid dysfunction. Therefore, given the accumulation of evidence, we here performed a systematic review and meta-analysis to address whether patients with RA are at higher risk of developing the four major thyroid disorders.

We performed a meta-analysis of the 29 included articles. Using the four types of thyroid dysfunction as categorical variables, the risk of thyroid dysfunction in RA patients increased to varying degrees, but the comprehensive analysis results also showed high heterogeneity. Subgroup analysis showed that the origin of control groups was the source of heterogeneity in the risk of hyperthyroidism, and study type was the source of heterogeneity for that of subclinical hypothyroidism in the correlation analysis.

When the control group consisted of healthy individuals, RA patients had a higher risk of hyperthyroidism compared with other non-RA patients. This suggests the need for further research into the relationship between thyroid dysfunction and other diseases. Such as the use of chemotherapy drugs as a systemic treatment for breast cancer increases the risk of thyroid dysfunction (52). In turn, a meta-analysis two years ago suggested that hyperthyroidism was associated with prostate, thyroid and breast cancers, compared with those with normal thyroid function (53). A Mendelian randomization study last year also provided further evidence of a causal link between thyroid disease and breast cancer risk (54). Subgroup analyses revealed that geographic region and year of publication were not the main sources of heterogeneity. However, subgroup analysis found that only studies conducted in Asia showed statistically significant differences between the RA group and the control group in terms of the prevalence of hyperthyroidism and subclinical hypothyroidism. In contrast, a subgroup analysis of hyperthyroidism and subclinical hyperthyroidism showed that the difference in the prevalence of these disorders between the RA and control groups was only statistically significant in the last decade compared with earlier studies. We speculated that this finding might be due to variations in detection device sensitivity, diagnostic thresholds, and iodine intake (**Table 2**) (49). The specific reasons need to be explored in future studies. In the sensitivity analysis of the primary variable, the combined results after excluding studies showed that the risk of developing thyroid dysfunction in RA patients remained significant, indicating that our findings were robust. However, in the sensitivity analysis of hyperthyroidism, after excluding the studies of Mahagna et al. (13) and Huang et al. (25), the combined OR value fluctuated greatly. Moreover, after excluding these two articles, heterogeneity was significantly reduced (P = 0.186, I^2^ = 30.3%). Therefore, they may be one of the main sources of heterogeneity. The retrospective cohort study by Huang et al. focused on the risk of hearing loss in patients with RA, not thyroid dysfunction. The relevant information could only be extracted from their baseline data. In addition, Huang et al.’s study included only newly diagnosed RA patients and excluded patients with a history of hearing loss, which may have confounded the data on the prevalence of thyroid dysfunction. Mahagna et al. used a cross-sectional study with inherent limitations and low methodological quality scores. Both studies used large databases and had large sample sizes compared to the other studies. However, irrespective of whether these two studies were included, the combined OR values suggested an increased risk of hyperthyroidism in RA patients. To provide more convincing evidence, more prospective studies are needed to more accurately assess the association between RA and thyroid disease. Although the funnel plot and Egger’s test indicated that there was indeed publication bias in meta-analysis of RA association with hypothyroidism, our combined results were proven to be robust by the trim-and-fill method, and that reporting bias did not significantly affect the results.

The underlying mechanisms of thyroid dysfunction in patients with RA are unclear (51). Thyroid dysfunction often occurs in autoimmune thyroid disease (AITD), particularly in Hashimoto’s thyroiditis and Graves’s disease. Moreover, it is now generally accepted that RA and AITD are caused by multiple factors, such as genetic susceptibility and environmental factors (55, 56). We found that patients with RA had a higher risk of developing hypothyroidism than other types of thyroid dysfunction. We hypothesized that the immunogenicity related to hyperthyroidism was lower than that related to hypothyroidism. The vast majority of hypothyroidism is caused by autoimmune diseases, such as Hashimoto’s thyroiditis, whereas autoimmune diseases lead to hyperthyroidism in relatively few cases and are influenced by age and environmental factors (57). A study using Mendelian randomization found a bidirectional causal relationship between RA and Graves’ disease, and good treatment and management of one may benefit the other (58). It is reasonable to speculate that thyroid dysfunction shares a common pathogenesis with RA (59). Exploring the co-pathogenesis pathway of these two diseases and appropriate application of drugs that can act on the co-pathogenesis targets may be an ideal way to prevent and treat co-diseases in the future (58, 60). In addition, a study by Raterman et al. also emphasized the need for careful drug selection in patients with RA complicated by autoimmune thyroiditis. They found that, in these patients, treatment with rituximab and l-thyroxine resulted in a transition to hyperthyroidism several months later (61).

This systematic review and meta-analysis still have the following limitations: First, the quality of the included studies was uneven. According to the NOS scale, 86.21% of the included studies were of medium and high quality, and 13.79% were of low quality. In order to include articles that were as fully compliant as possible, we had not excluded meeting abstracts and letters that provided important data, but which were not always sufficient in their description of detail. Most of these low-quality articles were meeting abstracts without available full text. This also affects, to some extent, the exploration of whether some important variables, such as the duration of RA and general sex distribution, are the main sources of heterogeneity. We performed a subgroup analysis of studies involving subclinical hyperthyroidism based on the mean age of RA patients and found that patients > 50 years had a significantly increased risk of having subclinical hyperthyroidism as compared with RA patients < 50 years (**eFigure 2**). The relationship between age and the occurrence of other types of thyroid dysfunction needs to be analyzed with more complete data.

Second, all included studies were observational studies, and the results of meta-analysis (62). However, sensitivity analyses indicated that our overall results were robust. Furthermore, using the trim-and-fill method, we confirmed that the combined results were not significantly affected by publication bias. Furthermore, due to the inclusion of observational studies, it is difficult to establish a causal relationship between RA and thyroid dysfunction, which needs to be confirmed by more prospective studies in the future.

Third, the results of meta-analyses with high heterogeneity should be interpreted with caution. Significant heterogeneity still could not be explained by subgroup analyses. Factors such as sex ratio, age, course of RA disease and other factors that were not statistically analyzed due to insufficient data may significantly influence the degree of risk of thyroid dysfunction in RA patients. In fact, gender and age are decisive risk factors for thyroid autoimmunity (15).

Fourth, the clinical criteria for assessing RA and thyroid dysfunction are inconsistent and should be addressed in future studies. In addition, because autoimmune diseases often co-occur, we excluded patients with SLE as controls, but the controls in this study were not all healthy individuals. The association between thyroid disease and SLE has been reported for over 50 years. We believe that using SLE as a control would have confounded the pooled results (17).

Fifth, a small number of reports were not included due to inability to find abstracts or full texts, and the results may have been partially affected. In addition, this study only discussed the risk of thyroid dysfunction in RA patients, and did not consider the incidence of thyroid diseases, such as Hashimoto’s thyroiditis and Graves’s disease. There are many causes of the four types of thyroid dysfunction, both primary and secondary, and their classification is complex. The lack of restriction on etiology is also part of the limitation of our meta-analysis. However, thyroid dysfunction is most common in autoimmune thyroid diseases, especially Graves’ disease and Hashimoto’s thyroiditis. Future studies are needed to more accurately compare the relationship between different thyroid diseases and RA (50, 57).

Our meta-analysis confirmed that RA patients have a high incidence of thyroid dysfunction, and routine biochemical testing of thyroid function in RA patients is reasonable. This not only enables the potential thyroid diseases of RA patients to be detected and treated in a timely manner, but more importantly, the therapeutic drugs for RA may aggravate the thyroid diseases, and the detection of thyroid function is helpful to clinicians for rational drug use (5). Common drugs used to treat RA, such as glucocorticoids (63) and leflunomide (64), interfere with thyroid function, whereas tumor necrosis factor inhibitors improve thyroid function in RA patients with hypothyroidism (65). It is suggested that the effects of various drugs related to the treatment of RA on thyroid function and the influence of thyroid dysfunction on the early treatment effect of RA should be studied more comprehensively (66). Compared with RA patients with negative thyroid autoantibodies, RA patients with positive thyroid autoantibodies had younger onset age and higher disease activity (67). Therefore, thyroid function should be tested regularly in younger RA patients. The close association between thyroid dysfunction and RA suggests a common pathogenic mechanism and potential therapeutic targets, which deserve further exploration.

## Conclusion

This meta-analysis revealed that RA patients have a higher prevalence of thyroid dysfunction, especially hypothyroidism. We conclude that it is reasonable to promote routine biochemical testing of thyroid function in patients with RA regardless of the presence or absence of obvious suspicious clinical manifestations, and vice versa. Our findings suggest that rheumatologists should screen RA patients for thyroid function and refer to endocrinologists to determine effective strategies for preventing and treating thyroid dysfunction. Furthermore, since RA may share a common pathogenesis with most AITDs that cause thyroid dysfunction, there may also be corresponding treatment options. More potential mechanisms and treatment options should be explored in future studies.

## Data availability statement

The original contributions presented in the study are included in the article/**Supplementary Material**. Further inquiries can be directed to the corresponding authors.

## Author contributions

YJL, ZC, and SL contributed to the conception and design of the systemic review. YJL and H-BM drafted and finalized the manuscript. ZC, H-BM, and SL revised the manuscript and provided critical advice on the content of the manuscript. All authors contributed to the article and approved the submitted version.

## Funding

This work was supported by grants from the Science and Technology Bureau of Quanzhou [grant number 2020CT003, 2017Z009]; the Natural Science Foundation of Fujian Province [grant number 2020J01219].

## Conflict of interest

The authors declare that the research was conducted in the absence of any commercial or financial relationships that could be construed as a potential conflict of interest.

## Publisher’s note

All claims expressed in this article are solely those of the authors and do not necessarily represent those of their affiliated organizations, or those of the publisher, the editors and the reviewers. Any product that may be evaluated in this article, or claim that may be made by its manufacturer, is not guaranteed or endorsed by the publisher.

## Abbreviations

CI, confidence interval; NOS, Newcastle–Ottawa Scale; OR, odds ratio; RA, rheumatoid arthritis; SLE, systemic lupus erythematosus; AITD, autoimmune thyroid disease; TSH, thyroid-stimulating hormone; T3, triiodothyronine; T4, thyroxine; fT3, free triiodothyronine; fT4, free thyroxine.
