# Supplementary material for: Association between rheumatoid arthritis and thyroid dysfunction: A meta-analysis and systematic review
Source: Front Endocrinol (Lausanne). 2022 Oct 13;13:1015516. doi: 10.3389/fendo.2022.1015516 (PMC9608862; doi:10.3389/fendo.2022.1015516)
Supplement: Supplementary file 1 [file Table_1.docx]

**eTable 1 Search strategy**

| PubMed | ("arthritis, rheumatoid"[MeSH Terms] OR "arthritis rheumatoid"[Title/Abstract] OR "rheumatoid arthritis"[Title/Abstract]) AND ("thyroid dysfunction"[Title/Abstract] OR ("thyroid diseases"[MeSH Terms] OR "thyroid diseases"[Title/Abstract] OR "disease thyroid"[Title/Abstract] OR "diseases thyroid"[Title/Abstract] OR "thyroid disease"[Title/Abstract]) OR ("hypothyroidism"[MeSH Terms] OR "hypothyroidism"[Title/Abstract] OR "Hypothyroidisms"[Title/Abstract] OR "thyroid stimulating hormone deficienc*"[Title/Abstract] OR "thyroid stimulating hormone deficienc*"[Title/Abstract] OR "tsh deficienc*"[Title/Abstract] OR "deficiency tsh"[Title/Abstract] OR "deficiency thyroid stimulating hormone"[Title/Abstract] OR "hormone deficiency thyroid stimulating"[Title/Abstract]) OR ("Hyperthyroidism"[MeSH Terms] OR "Hyperthyroidism"[Title/Abstract] OR "Hyperthyroid"[Title/Abstract] OR "Hyperthyroids"[Title/Abstract])) |
| --- | --- |
| Scopus | ( TITLE-ABS-KEY ( {thyroid dysfunction}  OR  {thyroid diseases}  OR  {disease, thyroid}  OR  {diseases, thyroid}  OR  {thyroid disease}  OR  {hypothyroidism}  OR  {hypothyroidisms}  OR  {thyroid-stimulating hormone deficienc*}  OR  {thyroid stimulating hormone deficienc*}  OR  {tsh deficienc*}  OR  {deficiency, tsh}  OR  {deficiency, thyroid-stimulating hormone}  OR  {hormone deficiency, thyroid-stimulating}  OR  {hyperthyroidism}  OR  {hyperthyroid}  OR  {hyperthyroids} )  AND  TITLE-ABS-KEY ( {Arthritis, Rheumatoid}  OR  {Arthritis, Rheumatoid}  OR  {Rheumatoid Arthritis} ) ) |
| Embase | #1 'thyroid disease'/exp  #2 'disease, thyroid':ab,ti OR 'thyroid abnormalities':ab,ti OR 'thyroid abnormality':ab,ti OR 'thyroid anomalies':ab,ti OR 'thyroid anomaly':ab,ti OR 'thyroid diseases':ab,ti OR 'thyroid disorder':ab,ti OR 'thyroid disorders':ab,ti OR 'thyroid dysfunction':ab,ti OR 'thyroid dysfunctions':ab,ti OR 'thyroid gland disease':ab,ti OR 'thyroid gland dysfunction':ab,ti OR 'thyroidal abnormality':ab,ti OR 'thyroidal anomaly':ab,ti OR 'thyroidal disease':ab,ti OR 'thyroidal disorder':ab,ti OR 'thyroidal dysfunction':ab,ti  #3 #1 OR #2  #4 'hypothyroidism'/exp  #5 'acute hypothyroidism':ab,ti OR 'hypothyreoidism':ab,ti OR 'hypothyreosis':ab,ti OR 'hypothyroidea':ab,ti OR 'hypothyroidosis':ab,ti OR 'hypothyrosis':ab,ti OR 'primary hypothyroidism':ab,ti OR 'thyroid deficiency':ab,ti OR 'thyroid gland failure':ab,ti OR 'thyroid insufficiency':ab,ti  #6 #4 OR #5  #7 'hyperthyroidism'/exp  #8 'feline hyperthyroidism':ab,ti OR 'hyperthyreoidism':ab,ti OR 'hyperthyreosis':ab,ti OR 'hyperthyroid function':ab,ti OR 'hyperthyroidea':ab,ti OR 'hyperthyroidosis':ab,ti OR 'thyroid gland hyperfunction':ab,ti OR 'thyroid hyperfunction':ab,ti OR 'thyroideal hyperfunction':ab,ti  #9 #7 OR #8  #10 #3 OR #6 OR #9  #11 'rheumatoid arthritis'/exp  #12 'arthritis deformans':ab,ti OR 'arthritis, rheumatoid':ab,ti OR 'arthrosis deformans':ab,ti OR 'beauvais disease':ab,ti OR 'chronic articular rheumatism':ab,ti OR 'chronic polyarthritis':ab,ti OR 'chronic progressive poly arthritis':ab,ti OR 'chronic progressive polyarthritis':ab,ti OR 'chronic rheumatoid arthritis':ab,ti OR 'disease, beauvais':ab,ti OR 'infantile rheumatoid arthritis':ab,ti OR 'inflammatory arthritis':ab,ti OR 'polyarthritis, primary chronic':ab,ti OR 'primary chronic polyarthritis':ab,ti OR 'rheumarthritis':ab,ti OR 'rheumatic arthritis':ab,ti OR 'rheumatic polyarthritis':ab,ti OR 'rheumatism, chronic articular':ab,ti  #13 #11 OR #12  #14 #10 AND #13 |
| Cochrane | #1 MeSH descriptor: [Hypothyroidism] explode all trees  #2 (hypothyroidism):ti,ab,kw OR (Hypothyroidisms):ti,ab,kw OR (Thyroid-Stimulating Hormone Deficienc*):ti,ab,kw OR (Thyroid Stimulating Hormone Deficienc*):ti,ab,kw  #3 (TSH Deficienc*):ti,ab,kw OR (Deficiency, TSH):ti,ab,kw OR (Deficiency, Thyroid-Stimulating Hormone):ti,ab,kw OR (Hormone Deficiency, Thyroid-Stimulating):ti,ab,kw  #4 #1 OR #2 OR #3  #5 MeSH descriptor: [Hyperthyroidism] explode all trees  #6 (Hyperthyroidism):ti,ab,kw OR (Hyperthyroid):ti,ab,kw OR (Hyperthyroids):ti,ab,kw  #7 #5 OR #6  #8 MeSH descriptor: [Thyroid Diseases] explode all trees  #9 (Thyroid Diseases):ti,ab,kw OR (Disease, Thyroid):ti,ab,kw OR (Diseases, Thyroid):ti,ab,kw OR (Thyroid Disease):ti,ab,kw  #10 #8 OR #9  #11 (thyroid dysfunction):ti,ab,kw  #12 #4 OR #7 OR #10 OR #11  #13 MeSH descriptor: [Arthritis, Rheumatoid] explode all trees  #14 (Arthritis, Rheumatoid):ti,ab,kw OR (Rheumatoid Arthritis):ti,ab,kw  #15 #13 OR #14  #16 #12 AND #15 |

**eTable 2 Egger’s test and Begg’s test**

|  | hyperthyroidism | subclinical hyperthyroidism | hypothyroidism | subclinical hypothyroidism |
| --- | --- | --- | --- | --- |
| Egger’s test | 0.223 | 0.065 | 0.002 | 0.187 |
| Begg’s test | 1.000 | 0.858 | 1.000 | 0.044 |

**eFigure** 1 **The trim-and-fill method for studies of hypothyroidism**

**
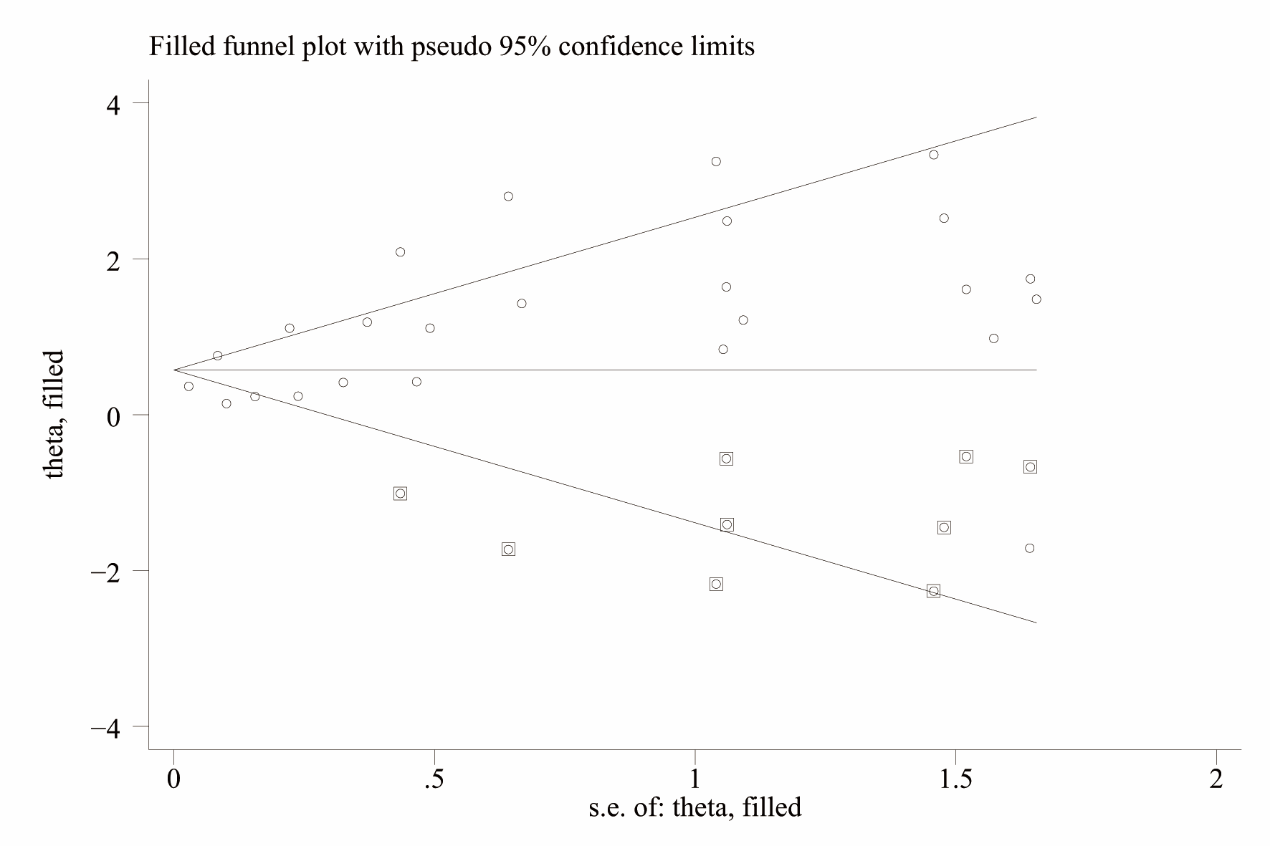
**

**eFigure 2** Age-related subgroup analysis of subclinical hyperthyroidism

**
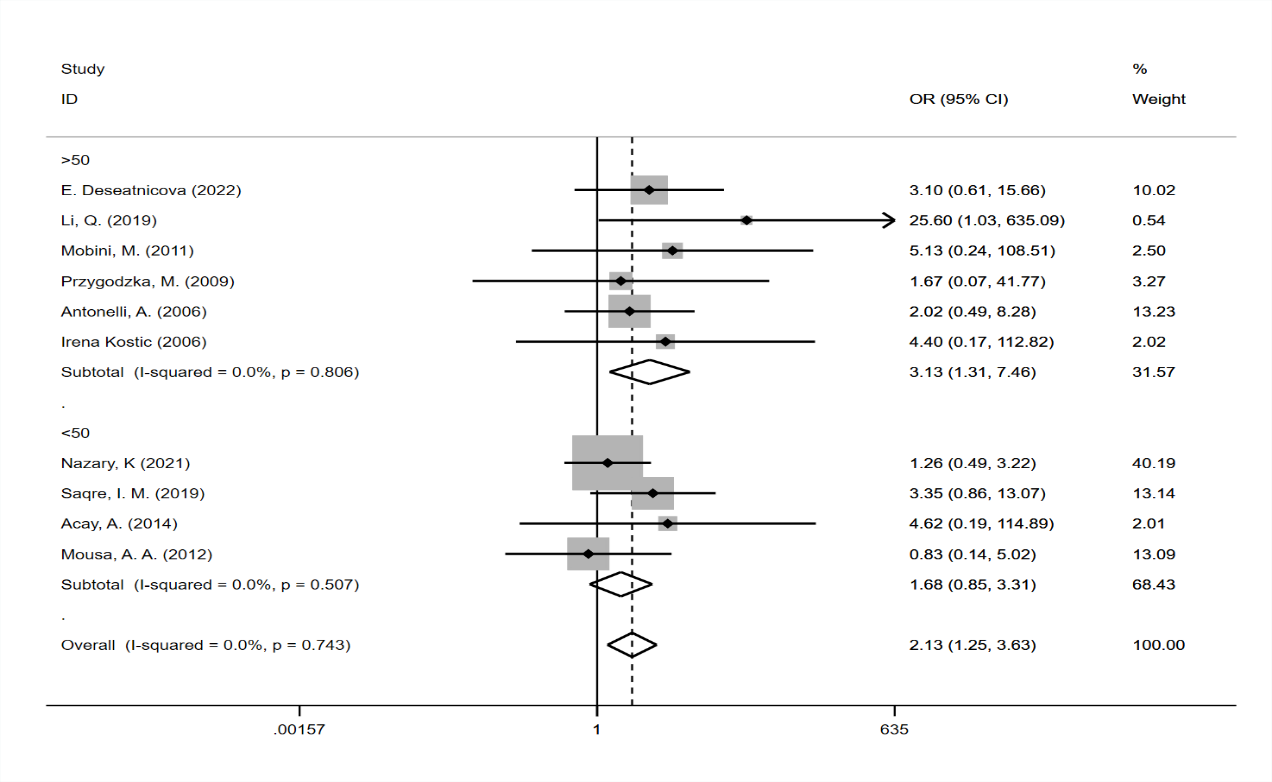
**
